# Supplementary material for: Identification of photoperiod-induced specific miRNAs in the adrenal glands of Sunite sheep (Ovis aries)
Source: Front Vet Sci. 2022 Jul 22;9:888207. doi: 10.3389/fvets.2022.888207 (PMC9354845; doi:10.3389/fvets.2022.888207)
Supplement: Supplementary file 1 [file Data_Sheet_1.ZIP › Supplementary file/miRDeep2 file.zip!/miRDeep2 file/Novel_Structure/SPLP42A3/Novel_639.pdf]

Provisional ID	: NC_019484.2_39179
Score total	: 2.7
Score for star read(s)	: -1.3
Score for read counts	: 0
Score for mfe	: 2.4
Score for randfold	: 1.6
Score for cons. seed	:
Total read count	: 13
Mature read count	: 12
Loop read count	: 0
Star read count	: 1

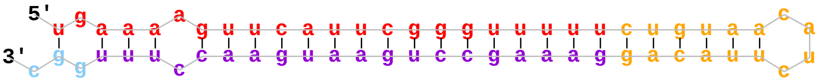

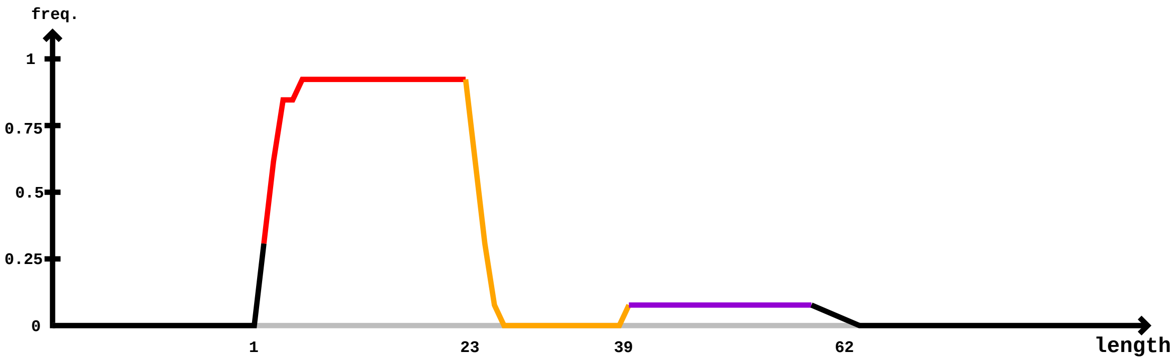

Mature Star

```
5'- uccccacauauuggguuggcugaaaaguucauucggguuuuucuguaacaucuuacaggaaagccugaaugaaccuuuggccaacccaauauuuccgaccaguugcaagggc  -3'   obs
    uccccacauauuggguuggcugaaaaguucauucggguuuuucuguaacaucuuacaggaaagccugaaugaaccuuuggccaacccaauauuuccgaccaguugcaagggc        exp
    .......(((((((((((((((((..((((((((((((((((((((((....)))))))))))))))))))))).)))))))))))))))))......((........))..  reads   mm   sample
    ....................ugaaaaguucauucggguuuuu......................................................................  4       0    seq
    .....................gaaaaguucauucggguuuuu......................................................................  4       0    seq
    ......................aaaaguucGuucggguuuuucu....................................................................  2       1    seq
    ......................aaaaguucauuAggguuuuucu....................................................................  1       1    seq
    ........................aaguucauucggguuuuucug...................................................................  1       0    seq
    ..........................................................gaaaAccugaaugaaccuuu..................................  1       1    seq
```
